# Chitosan-Starch Films with Natural Extracts: Physical, Chemical, Morphological and Thermal Properties

**DOI:** 10.3390/ma11010120

**Published:** 2018-01-12

**Authors:** Jessica I. Lozano-Navarro, Nancy P. Díaz-Zavala, Carlos Velasco-Santos, José A. Melo-Banda, Ulises Páramo-García, Francisco Paraguay-Delgado, Ricardo García-Alamilla, Ana L. Martínez-Hernández, Samuel Zapién-Castillo

**Affiliations:** 1 Tecnológico Nacional de México-Instituto Tecnológico de Ciudad Madero, Centro de Investigación en Petroquímica, Prolongación Bahía de Aldair, Ave. De las Bahías, Parque de la Pequeña y Mediana Industria, Altamira, Tamaulipas 89600, Mexico; yukino_85@hotmail.com (J.I.L.-N.); melobanda@yahoo.com.mx (J.A.M.-B.); uparamo@itcm.edu.mx (U.P.-G.); ricardogarcia.alamilla@yahoo.com.mx (R.G.-A.); szc_0190@hotmail.com (S.Z.-C.); 2 Tecnológico Nacional de México-Instituto Tecnológico de Querétaro, -División de Estudios de Posgrado e Investigación, Av. Tecnológico s/n esquina Gral. Mariano Escobedo, Centro Histórico, Querétaro, Querétaro 76000, Mexico; cylaura@gmail.com (C.V.-S.); almh72@gmail.com (A.L.M.-H.); 3 Centro de Investigación en Materiales Avanzados, S. C., Departamento de Materiales Nanoestructurados, Miguel de Cervantes 120, Complejo Industrial Chihuahua, Chihuahua, Chihuahua 31136, Mexico; francisco.paraguay@cimav.edu.mx

**Keywords:** polysaccharides, composite films, chitosan-starch, natural antioxidants, film properties

## Abstract

The aim of this study is to analyze the properties of a series of polysaccharide composite films, such as apparent density, color, the presence of functional groups, morphology, and thermal stability, as well as the correlation between them and their antimicrobial and optical properties. Natural antioxidants such as anthocyanins (from cranberry; blueberry and pomegranate); betalains (from beetroot and pitaya); resveratrol (from grape); and thymol and carvacrol (from oregano) were added to the films. Few changes in the position and intensity of the FTIR spectra bands were observed despite the low content of extract added to the films. Due to this fact, the antioxidants were extracted and identified by spectroscopic analysis; and they were also quantified using the Folin-Denis method and a gallic acid calibration curve, which confirmed the presence of natural antioxidants in the films. According to the SEM analysis, the presence of natural antioxidants has no influence on the film morphology because the stretch marks and white points that were observed were related to starch presence. On the other hand, the TGA analysis showed that the type of extract influences the total weight loss. The overall interpretation of the results suggests that the use of natural antioxidants as additives for chitosan-starch film preparation has a prominent impact on most of the critical properties that are decisive in making them suitable for food-packing applications.

## 1. Introduction

Chitosan is a cationic polysaccharide (obtained from the deacetylation of chitin, coming from the shells of crustaceans and insect exoskeletons) that is a suitable packaging material due to its biocompatibility, biodegradability, non-toxicity, film-forming and antimicrobial properties. This polymer is currently used in food packaging, agriculture, biomedical science and cosmetics. It can be used as a transparent film or coating to improve food quality and to extend its shelf life. Chitosan is one of the most abundant natural biopolymers with proven antimicrobial, antioxidant, and antifungal/viral properties [1,2,3].

Starch is a biodegradable and thermoplastic polymer formed mainly by two polysaccharides: amylose and amylopectin. In recent research, this polymer is considered to be a promising agent for applications such as food packaging due to its biodegradability and flexibility [3]. In this study, starch from rice was used, which is a less studied starch source. According to Jiang et al. [4], the starch from rice is convenient for preparing chitosan-starch films because of its relatively small particle size (2–8 µm) and its hypoallergenic quality, which make it an appropriate material for food industry utilization. 

On the other hand, antioxidants such as anthocyanins, betalains, thymol, carvacrol and resveratrol have attracted attention because of their recently discovered antimicrobial properties. Anthocyanins are natural, water-soluble colorants obtained from plants, flowers and fruits having orange, pink, red, violet and blue colors. Also, they have been demonstrated to be useful for the prevention of several diseases. Their stability is influenced by several factors such as pH, storage temperature, chemical structure, concentration, light, oxygen, solvents and metals [5]. In this research, natural anthocyanins extracts obtained from cranberry (*Vaccinium oxycoccus*), blueberry (*Vaccinium myrtillus*) and pomegranate (*Punica granatum*) were analyzed.

Betalains are pigments found in red–violet (betacyanins) or yellow (betaxanthins) plants, vegetables and fruits that play a key role in human health. Natural extracts containing betalains (betacyanins) from beetroot (*Beta vulgaris*) and pitaya (*Hylocereus undatus*) were investigated in this research. Beetroot contains two major pigments: betanin (a red betacyanin) and vulgaxanthine I (a yellow betaxanthin). They have antioxidant and antimicrobial properties. Pitaya is rich in betacyanins such as betanin, phyllocactin, hylocerenin and their isomers [6]. The stability of betalains is influenced by many factors such as temperature (the most important factor), pH, water activity, light, the presence or absence of oxygen, and enzymatic action [7]. 

Thymol and carvacrol are monoterpenes found in spices such as thyme and oregano. They are highly appreciated because of their antioxidant and antimicrobial activity. Owing to their phenolic nature, they are highly efficient as bactericidal agents [8,9]. In this study, natural extracts of thymol and carvacrol obtained from oregano (*Origanum vulgare*) were evaluated.

Resveratrol is a polyphenol produced by plants such as grapes, blueberries, mulberries, cranberries and peanuts, in response to any physical damage, UV light, and fungi presence [10]. In recent years, this compound has been the object of several studies because of its advantageous effects on human health and its antioxidant activity [11]. In this work, resveratrol extracted from grape (*Vitis vinifera*) was used for the film preparation.

The use of anthocyanins improves the performance of chitosan films in food applications according to Ge et al. [12]. Furthermore, as stated by the results of the investigation of Berizi et al. [13], the addition of pomegranate peel extract to a chitosan solution prevents the oxidation of fats and proteins of rainbow trout, while enhancing the chemical, antimicrobial and textural properties of a packaging film that was used to preserve the rainbow trout for a storage period of up to six months. According to Avila-Sosa et al. [14], the combination of chitosan and oregano is becoming an appropriate material to substitute for some of the synthetic polymers used in food packaging applications. This kind of biomaterial offers desirable properties such as antimicrobial, mechanical and barrier properties. 

The present article reports the synthesis and characterization of chitosan-starch films modified by the addition of cranberry, blueberry, beetroot, pomegranate, oregano, pitaya and resveratrol aqueous extracts. The influence of these natural antioxidant extracts on the properties of chitosan-starch films was evidenced through an innovative method aiming to enhance the chemical, morphological and thermal properties of the films, as well as their coloration and apparent density. The method proposed in this research is simple, effective and inexpensive because it implies the addition of organic, non-toxic and naturally available extracts, reducing costs, the use of solvents and time. These materials also presented improved antimicrobial, mechanical and optical properties as shown in a previously reported investigation [15]. These results were correlated with those obtained in that research in order to have a more comprehensive understanding of the influence of natural extracts in the chitosan-starch blend. The overall performance of the modified films suggests that most of these materials are promising candidates for further food packaging applications. Improving these features is important because it allows the proposed materials to become more effective than unmodified chitosan-starch film against the two main causes of food decomposition: the presence of micro-organisms and the degradation of lipids.

## 2. Results

### 2.1. Evaluation of Films Coloration (Visual and Image Analyzer Software)

**Figure 1:**
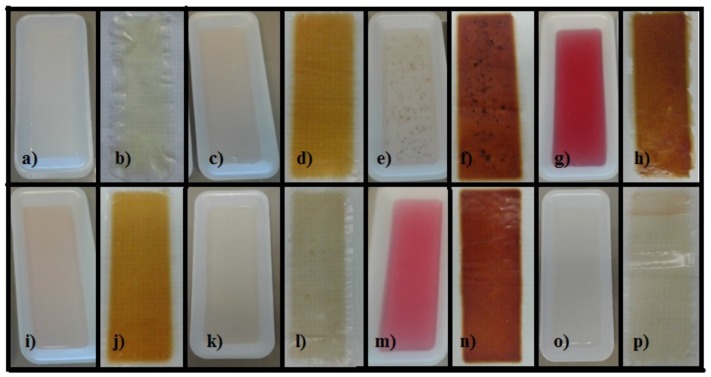
Coloration of films 15: (**a**) QS2 day 1; (**b**) QS2 day 15; (**c**) QSA2 day 1; (**d**) QSA2 day 15; (**e**) QSAm2 day 1; (**f**) QSAm2 day 15; (**g**) QSB2 day 1; (**h**) QSB2 day 15; (**i**) QSG2 day 1; (**j**) QSG2 day 15; (**k**) QSO2 day 1; (**l**) QSO2 day 15; (**m**) QSP2 day 1; (**n**) QSP2 day 15; (**o**) QSR2 day 1; and (**p**) QSR2 day 15.

In Table 1, film characteristics such as color, flexibility and translucency are indicated. As shown in Figure 1, most of the films with natural extracts were colored due to the presence of extracts as well as their antioxidants and sugars, and also because of their interaction with the environment. For more details about color changing, see Section 3.1. To see films’ nomenclature, see footnote of Table 1. The film QS2 is translucent and flexible. The film QSA2 is translucent and more flexible than QS2. The film QSAm2 is translucent and more flexible than QS2. The film QSB2 is translucent and more flexible than QS2. The film QSG2 is translucent and more flexible than QS2. The film QSO2 is translucent and it was the most flexible one. The film QSP2 is translucent and less flexible than QS2. The higher the extract concentration, the more brittle it becomes. The film QSR2 is translucent and more flexible than QS2. In Table 1, the average RGB values at day 1 and 15 are indicated. It was observed that the RGB values diminish from day 1 to 15, which means that the films underwent a change in coloration, tending to become darker. This is confirmed by seeing the photographs in Figure 1. As displayed in Table 1, in most of the cases the films presented several variations of yellow, red and brown colors. This is confirmed by the color theory: RGB values around 165, 42, 42 correspond to brown, while maroon has RGB values around 128, 0, 0; sienna has RGB values of 160, 82, 45 and saddle brown is formed by RGB values 139, 69, 19. Rosy brown corresponds to RGB values of 188, 143, 143; dark goldenrod has RGB values of 184, 134, 11, while RGB values of 178, 34, 34 correspond to firebrick; all of these colors are variations of yellow, red or brown. If the experimental RGB values of the samples are compared with those parameters, the observation of yellowish, reddish or brownish colors are correctly validated. Therefore, the comparison between the theoretical and the real values confirms the colors that were observed at days 1 and 15. The films presented similar coloration with different intensity (more details about color theory are explained in Section 4.3) [16].

### 2.2. Apparent Density

The apparent density results are indicated in Table 2. The changes in this parameter are related to the porosity, filtration capacity and aeration of the materials. The lower the apparent density, the higher the values of the cited properties [17]. 

### 2.3. Fourier Transform Infrared Spectroscopy (FTIR)

**Figure 2:**
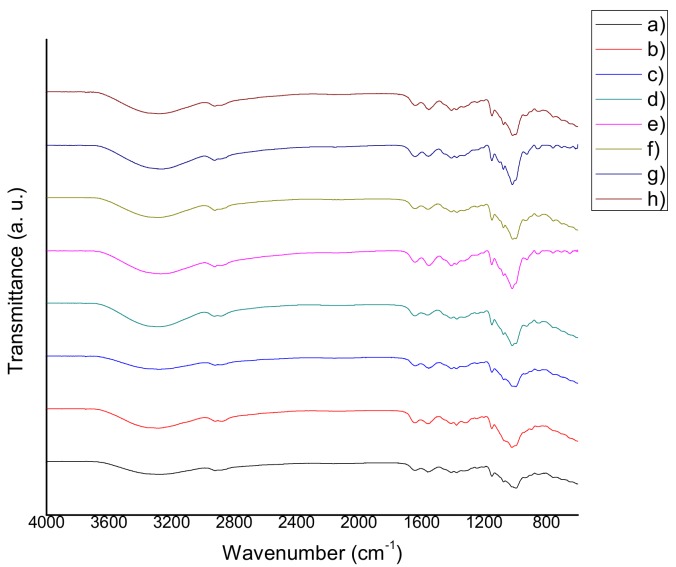
FTIR spectra of (**a**) QS2 vs. (**b**) QSA0.5; (**c**) QSAm0.5; (**d**) QSB0.5; (**e**) QSG0.5; (**f**) QSO0.5; (**g**) QSP0.5 and (**h**) QSR0.5.

**Figure 3:**
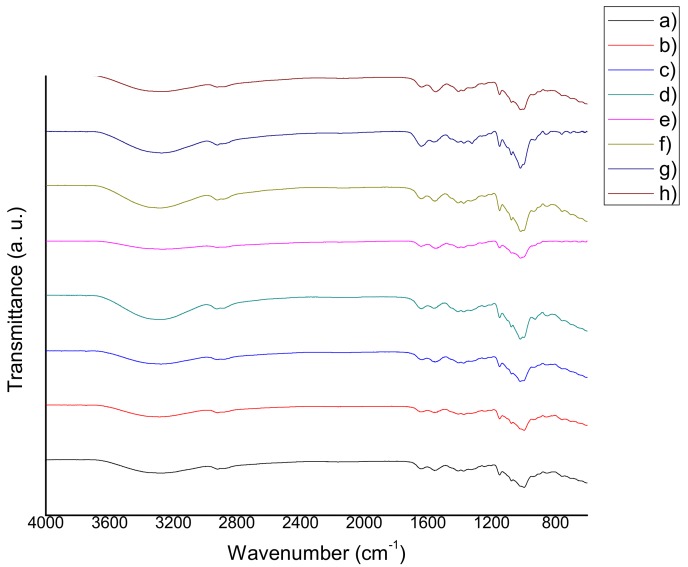
FTIR spectra of (**a**) QS2 vs. (**b**) QSA2; (**c**) QSAm2; (**d**) QSB2; (**e**) QSG2; (**f**) QSO2; (**g**) QSP2; and (**h**) QSR2.

**Figure 4:**
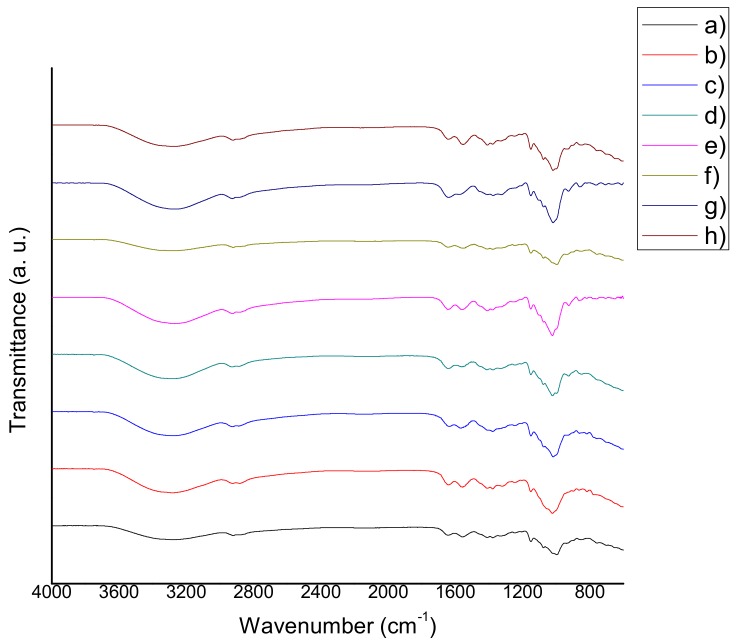
FTIR spectra of (**a**) QS2 vs. (**b**) QSA5; (**c**) QSAm5; (**d**) QSB5; (**e**) QSG5; (**f**) QSO5; (**g**) QSP5; and (**h**) QSR5.

The FTIR spectra of chitosan-starch films are shown in Figure 2, Figure 3 and Figure 4. The following characteristic peaks were identified: a wide band between 3390 cm^−1^ and 3180 cm^−1^, attributed to stretching of –OH bonds of chitosan and starch, as well as the hydrogen bonds occurring as a result of their interaction with solvents; in this same region, the peak corresponding to the stretching of –NH bonds of chitosan was observed. At 2950–2915 cm^−1^ the peak that corresponds to –CH bonds of chitosan, appeared. At 1660–1650 cm^−1^, a characteristic peak of the C=O stretching was found. At 1580–1570 cm^−1^, the corresponding peak of the bending of –NH of chitosan can be seen (in most of the cases, a more intense peak was observed at 1580–1570 cm^−1^, which is associated with the deacetylation process and indicates the prevalence of NH_2_ groups). At 1370–1350 cm^−1^, the peaks attributed to the C–N stretching of amide III group were found. The presence of the C–O–C bond was observed between 1160 cm^−1^ and 1118 cm^−1^. At 1098–1080 cm^−1^, a peak that corresponds to the stretching of C–O bond was located. At 1040–970 cm^−1^, a peak corresponding to the stretching of C–O–C bond was observed. Finally, peaks between 900 cm^−1^ and 600 cm^−1^ attributed to the C–H and C=C bonds were observed [18,19,20,21]. The presence of aqueous extracts could induce changes in the transmittance percentage and intensity of these peaks. 

### 2.4. Determination of Antioxidant Capacity (Reducing Capacity) of the Films

#### 2.4.1. Qualification of Natural Antioxidants

**Figure 5:**
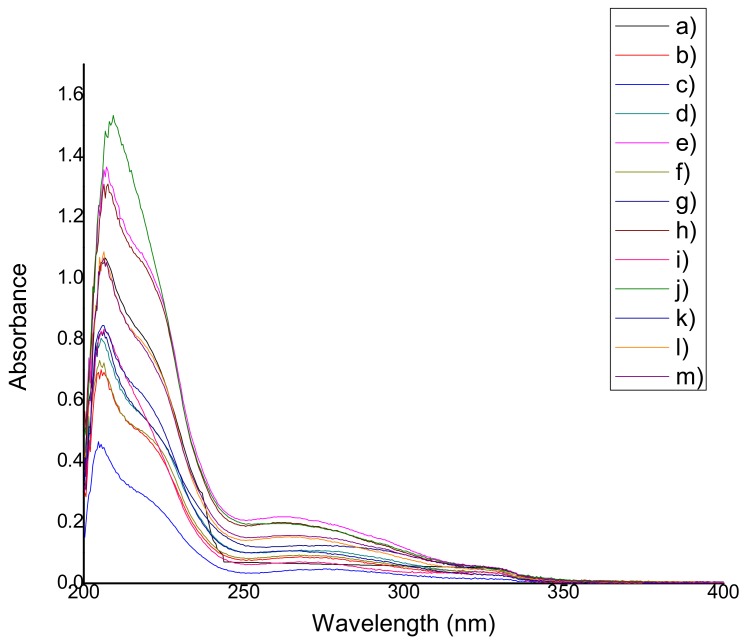
UV spectrum of (**a**) QS2; (**b**) QSA0.5; (**c**) QSA2; (**d**) QSA5; (**e**) QSAm0.5; (**f**) QSAm2; (**g**) QSAm5; (**h**) QSB0.5; (**i**) QSB2; (**j**) QSB5; (**k**) QSG0.5; (**l**) QSG2; and (**m**) QSG5.

**Figure 6:**
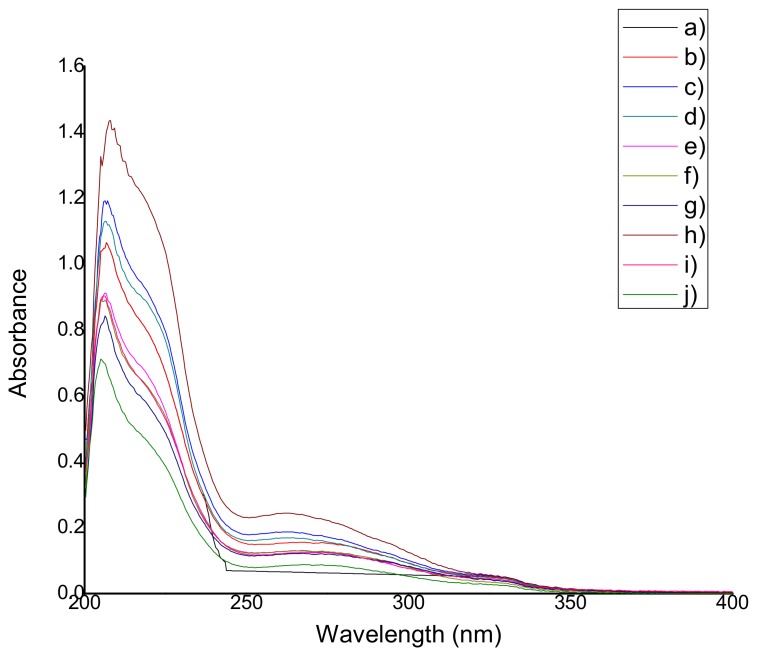
UV spectrum of (**a**) QS2; (**b**) QSO0.5; (**c**) QSO2; (**d**) QSO5; (**e**) QSP0.5; (**f**) QSP2; (**g**) QSP5; (**h**) QSR0.5; (**i**) QSR2; and (**j**) QSR5.

The UV spectra (obtained from the average of three tests) of all the films containing natural extracts and the control film QS2 are shown in Figure 5 and Figure 6. For qualification of the antioxidants, it was considered that the characteristic band of anthocyanins appears around 280 nm [22], thymol and carvacrol have a characteristic band around 270 nm [23], and polyphenols (betalains and resveratrol are part of this group as well) are identified in the 280–350 nm region [24]. Ethanol instead of methanol was used because of its lower toxicity and because there are no significant differences among absorbance values according to other investigations [25].

Table 3 shows the wavelength at which the antioxidants characteristic bands were found for each film. In all cases, a hypochromic shift was observed due to the presence of certain functional groups such as carboxyl, hydroxyl, and phenolic groups. It may also have occurred because of the increased polarity of the solvent and the acidity of the medium. There is no strong relationship between the extract concentration used and the absorbance value.

#### 2.4.2. Antioxidant Capacity (Reducing Capacity)

**Figure 7:**
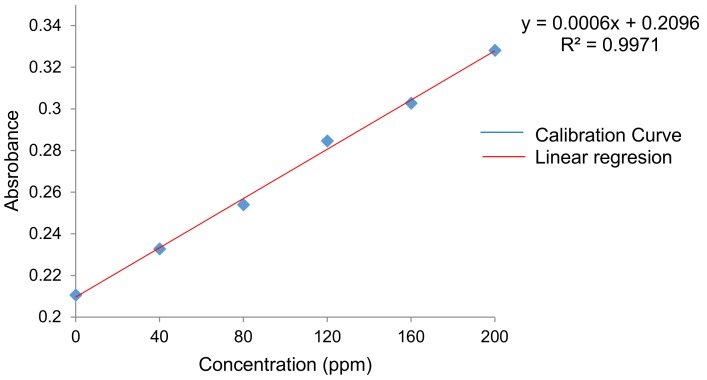
Calibration curve of gallic acid.

The calibration curve of gallic acid is shown in Figure 7. The average antioxidant capacity of each film is indicated in Table 4 and does not seem to differ much from one sample to another. Therefore, an ANOVA was conducted for properly measuring the significance of the slight differences of this property. 

An unbalanced multifactorial analysis of variance (ANOVA) was performed in order to test the statistical significance of the differences in the antioxidant capacity among the studied samples, using Minitab 16. In this analysis, the typical significance level of α = 0.05 was used. Three factors were evaluated: type of antioxidant (4 levels: anthocyanins, betalains, resveratrol, thymol-carvacrol), source of the antioxidant represented by the extract nested into the antioxidant factor (7 levels: blueberry, cranberry, pomegranate, beetroot, pitaya, oregano, resveratrol) and the content of extract, expressed as a weight percent (3 levels: 0.5%, 2%, 5%). 

According to the p-values obtained in the ANOVA analysis, significant differences in the mean antioxidant capacities occurred due to almost all the factors and their interactions, except for the extracts (*p*-value = 0.336). In fact, the mean antioxidant capacity of six out of the seven levels of the extract factor equaled 0.2100 mg/mg, whereas only one level possessed a different mean antioxidant capacity, corresponding to thymol-carvacrol, and according to its *p*-value that variation would most likely be the consequence of natural randomness. On the other hand, the *p*-value of the rest of the factors was lower than the significance level of the test, which implies that the subtle differences among the mean antioxidant capacities of the samples are explained by the variation in the type (*p*-value < 0.0005) and content (*p*-value = 0.009) of antioxidants. Furthermore, the method proved that the type and content (*p*-value = 0.001), as well as the source and content (*p*-value = 0.021) of the antioxidants interact to produce major differences in the mean antioxidant capacity, which means that these factors not only produce isolated effects on the mean antioxidant capacities of the films, but also, they depend on each other. It is important to point out that even though the extracts by themselves, do not cause significant variation in the mean antioxidant capacity, this factor in combination with the weight percent actually exerts a statistically noticeable effect on the antioxidant capacity. With the aim of identifying those particular levels of each factor that generate the observed differences in the mean antioxidant capacity, the Tukey method was chosen as a post hoc test for further analysis. From the results of the Tukey test, the antioxidants that provoke the most important variations in antioxidant capacity are the thymol-carvacrol, betalains and anthocyanins. Regarding the content of extract, the Tukey test showed that the significant mean differences come from the change from 0.5% to 2% of extract and, interestingly, from the same statistical perspective, increasing the extract content to 5% does not have any significant effect on the antioxidant capacity of the films.

According to the ANOVA results, the most important variations in antioxidant capacity correspond to the oregano extract, the betalains from beetroot and the three sources of anthocyanins (cranberry, blueberry and pomegranate). The antioxidant capacity might be correlated to the antimicrobial activity of the films, given that in a previously reported study the addition of extracts, especially at concentrations of 2% and 5% *v*/*v*, enhanced antimicrobial activity of chitosan-starch films, except for the case of pitaya extract, whose most suitable concentration was 0.5% *v*/*v*. The antimicrobial activity of the films with extracts containing anthocyanins and betalains is a little bit higher than the rest, because these extracts also contain sugars and vitamin C that stabilize the antioxidants and upgrade their antimicrobial activity [15]. 

### 2.5. Scanning Electronic Microscopy (SEM)

**Figure 8:**
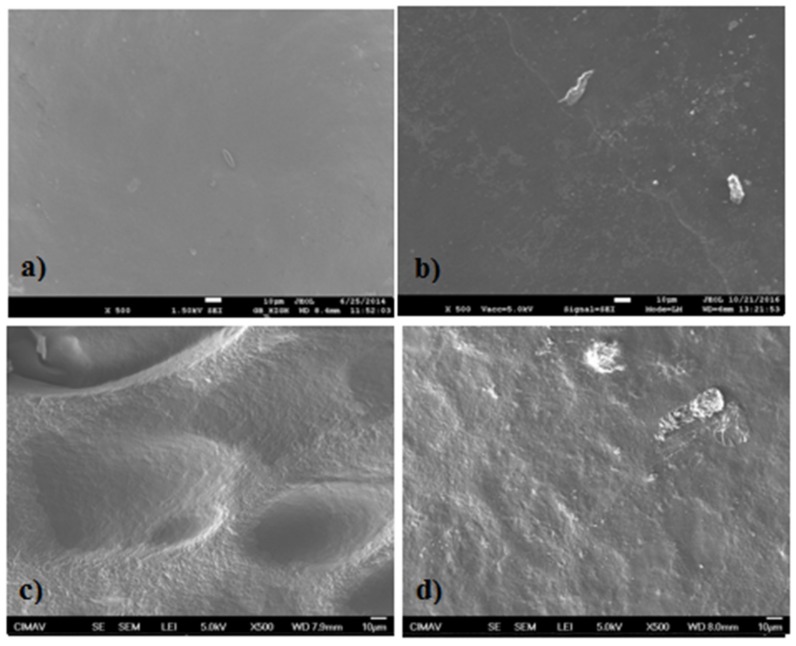
Scanning electronic micrograph (SEM) of (**a**) QS2; (**b**) QSA5; (**c**) QSAm5; and (**d**) QSB5 at 500×.

**Figure 9:**
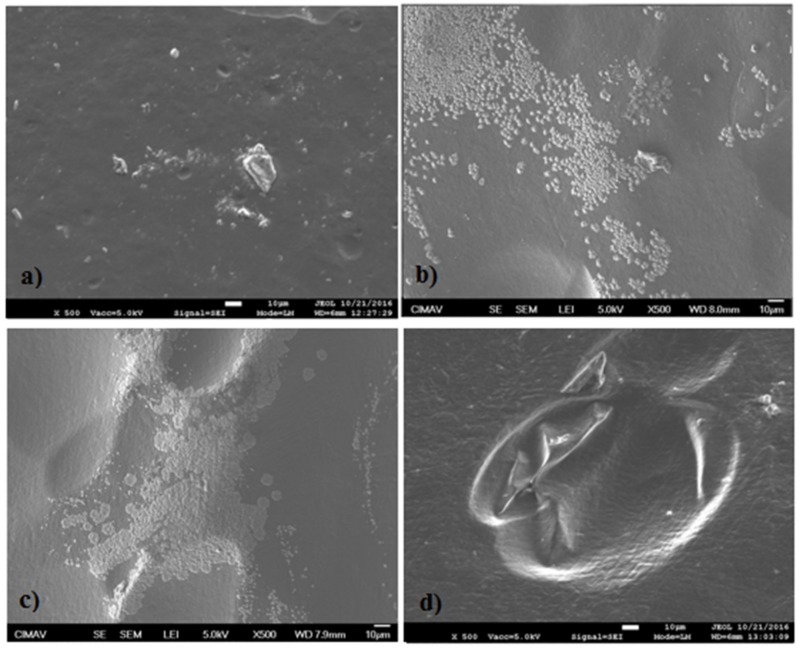
Scanning electronic micrograph (SEM) of (**a**) QSG5; (**b**) QSO5; (**c**) QSP0.5; and (**d**) QSR5 at 500×.

In Figure 8 and Figure 9, the micrographs (at magnification of 500×) obtained from some samples with extracts and the QS2 film are shown. In Figure 8a, QS2 showed a homogeneous surface without phase separation between the polysaccharides, indicative of a good interaction between the components. In Figure 8b, QSA5 showed an almost homogeneous surface; the presence of ribbed lines or streaks and white spots associated with the presence of starch is observed. In Figure 8c, multiple oval convexities, streaks and white spots are observed in QSAm5 film. Figure 8d shows that QSB5 has an almost homogeneous surface with streaks and white spots. 

In Figure 9a, QSG5 shows an almost homogeneous surface with streaks and white spots. Figure 9b reveals that QSO5 has few convexities and some agglomeration because of the presence of starch. In Figure 9c, few convexities and some starch agglomeration in QSP0.5 are observed. Figure 9d presents the morphology of QSR5, whose shapes resemble “Brussels sprouts”, as well as several streaks and tiny white dots (attributed to the presence of starch granules). The surface looks, in certain places, almost smooth at greater magnifications (these magnifications are not shown). 

### 2.6. Thermogravimetric Analysis (TGA)

**Figure 10:**
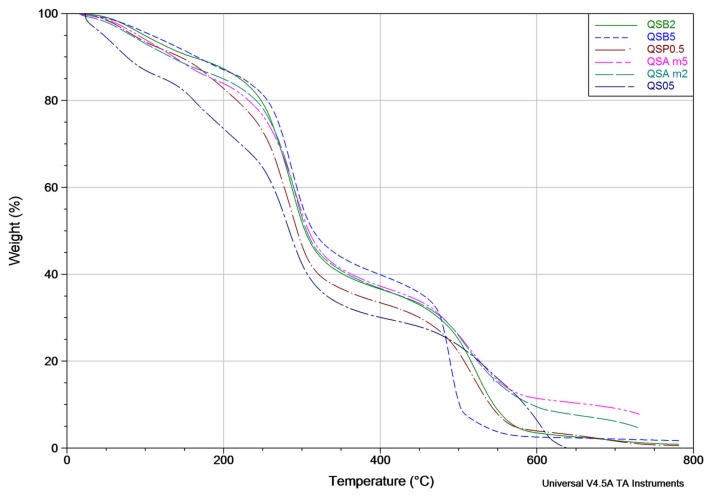
Thermograms of QSAm2, QSAm5, QSB2, QSB5 and QSO5 films.

The TGA curves for QSAm2, QSAm5, QSB2, QSB5, QSO5 and QSP0.5 films are shown in Figure 10. In each TGA curve, several weight losses or thermal events are evidenced. The first weight loss, which takes place from the initial temperature to approximately 135 °C, is associated to the removal of moisture and volatile materials; even the degradation of the antioxidants should occur at the end of this weight loss. The second weight loss, between 135 °C and 320 °C, occurs as a result of the decomposition of the chitosan amino units and the plasticization of starch, as well as the decomposition of glycerol (which occurs at 290 °C). The third loss occurs between 320 °C and above 400 °C, and is related to the decomposition of the –CH_2_OH group. The total degradation of the chitosan and starch cyclic structures arises at 600 °C. 

The films QSAm2 and QSAm5, in comparison with the rest of the samples, present a greater displacement at the end of the curve, which could indicate a greater plasticization in the materials that contain blueberry. All the curves have the same initial shape caused by the acetylation of the material, except for QSO5 [26,27,28,29,30]. The Table 5 displays the weight loss percentages of each film.

The QSAm2, QSAm5 and QSO5 films experienced the lowest total weight loss and they correspondingly showed the highest antimicrobial activity against mesophilic aerobic and coliform bacteria and fungi among all the chitosan-starch films, as presented in a prior research [15]. It should be noted that, regarding the % weight loss at 135 °C, the results suggest that the antioxidants coming from beetroot, blueberry and pitaya are more thermally stable than thymol and carvacrol from oregano. 

## 3. Discussion

### 3.1. Evaluation of Films Coloration

There is a significant difference between the coloration of the films containing cranberry, blueberry, beetroot, pomegranate and pitaya, due to the presence of antioxidants such as anthocyanins and betalains, which are also natural pigments. The coloration change in day 15 is visual evidence of the color degradation that takes place in the films because of the increase in pH values from day 1 to 15 [15] and because of their interaction with the environment, especially with oxygen and light. The presence of light triggers the degradation of antioxidants, and moreover, the combination of oxygen and vitamin C (present in cranberry, blueberry, pomegranate and pitaya) can enhance the degradation of antioxidants. However, the presence of sugars is a major stabilizing factor for antioxidants [31,32,33,34]. In the case of the oregano films, they did not suffer any noticeable change in coloration, because thymol and carvacrol are colorless [35]. Regarding the resveratrol films, changes in their coloration were observed (the whitish component in combination with grape colorants, produces a film with a very light brown color) because resveratrol is unstable to light, and it is also affected by changes in pH [36]. Besides the effect of light and oxygen, a relationship between film pH increment and color changing was noticed, with values oscillating between 4.34 and 6.14 [15], making them suitable for antioxidant activity given that the anthocyanins are stable at pH 3–7 and the betalains at pH 5–6 [35,37].

### 3.2. Apparent Density 

The addition of cranberry and blueberry to the chitosan-starch films led to an increase in their density, while beetroot, pomegranate, oregano, pitaya and resveratrol (except for QSB0.5, QSG0.5 and QSG5), decreased this property. The addition of natural extracts caused most of the films to become more porous than QS2, which results in enhancing their permeability, that is, the transmission of a fluid, such as water vapor and oxygen, because this property is directly related to the porosity [38,39]. The film permeability is closely related not only to the apparent density values but also to the film thickness, which, in turn, has proved to be associated with antimicrobial activity against aerobic mesophilic, total coliforms and fungi. For instance, the addition of 2% and 5% (*v*/*v*) of extracts considerably improved the antimicrobial activity of most of the tested films in a previous work, except for the case of pitaya, whose best results were those corresponding to the lower quantity of extract (0.5% *v*/*v*) [15]. From this analysis it can be inferred that the films with higher apparent density values may show better antimicrobial activity as a result of an improved oxygen barrier effect and a reduction of moisture transfer due to their lower porosity. This property might also enable the migration of inhibitory compounds such as antioxidants and amino groups of chitosan to the wall cell of micro-organisms, and because they need these compounds to survive; if they are isolated from these components, they will not grow properly [40]. 

### 3.3. Fourier Transform Infrared Spectroscopy (FTIR)

According to several authors, the molecular weight of chitosan has no influence on the absorption bands of the films [41,42,43,44,45,46]. However, the films that were modified with natural extracts in this study, exhibited shifts in their characteristic peaks to lower frequencies, in comparison with QS2. This behavior can be explained because of the non-covalent interactions among the functional groups of chitosan, starch, glycerol and those of the natural extracts (antioxidants). The same behavior was found and discussed in the research of Qin et al. [47]. Reduction in the % transmittance of the films containing natural extracts can be related to the decrement of transparency and the increment of opacity revealed by the optical properties tests, because the addition of natural extracts produced films with a more intense color. Wider transition zones were present in tan delta curves, which may be indicative of a possible reaction between the functional groups of the extracts (antioxidants) and the matrix of the samples. This behavior may also be explained either because of the fact that the film blend is more complex than QS2, or because of a possible decomposition of antioxidants that takes place between 50 °C and 100 °C. These phenomena can explain the large band at 3420 cm^−1^ (stretching –OH) in the infrared spectra of the films [15]. There are no observable major differences between the characteristic peaks of the spectra obtained in this study, and those published by the researchers referred to. The films with natural extracts presented a wider band around 3500–3200 cm^−1^, in comparison to QS2, which implies the presence of natural extract compounds with –OH bonds such as water, saccharides, carboxylic acids, antioxidants, etc.

### 3.4. Determination of Antioxidant Capacity (Reducing Capacity) of the Films

From the obtained results, it was demonstrated that the most important factor is not the antioxidant capacity value, but the type of antioxidant used, because it is what truly affects the antimicrobial, mechanical, optical and thermal properties of the films [15]. It was observed that anthocyanins and betalains achieved the best results; which may be an outcome of their large stability to environmental conditions [21,37,47].

### 3.5. Scanning Electronic Microscopy (SEM)

As can be deduced from the micrographs presented in Section 3, the chitosan and starch have a relatively good interfacial adhesion [46,48,49]. The film morphology is not directly related neither to the antimicrobial activity nor to the optical properties of the films [15]. 

### 3.6. Thermogravimetric Analysis (TGA)

According to the results of the first weight loss, the natural extracts from beetroot, cranberry and pitaya are more stable than that coming from oregano. This is consistent with the fact that anthocyanins and betalains possess higher stability to temperature variations in comparison with thymol and carvacrol. A similar behavior was observed by Espitia et al. [50], who found that the addition of apple skin phenols and acai increases thermal stability of edible films. Moreover, Altiok et al. [51] observed that the addition of thyme oil affects thermal stability; the higher the concentration of oregano, the lower the weight loss of chitosan films. 

## 4. Materials and Methods

### 4.1. Materials

The materials used for the film synthesis are detailed as follows: chitosan (medium molecular weight and a degree of deacetylation of 85%) and rice starch were purchased from Sigma-Aldrich (Toluca, Edo. Mex., Mexico), glacial acetic acid (99.9% purity) and glycerol (99.7% purity) were purchased from Fermont (Monterrey, NL, Mexico). Natural antioxidants were obtained from raw fruits: cranberry, blueberry, beetroot and pitaya, from dry oregano, commercial cranberry and pomegranate juices which were purchased in a local market. Resveratrol capsules were purchased from General Nutrition Centers (Pittsburgh, PA, USA). The extracts of blueberry, beetroot and pitaya were obtained from raw fruit pulp at 25 °C and were filtered prior to their addition in the chitosan–starch–glycerol blend. An aqueous extract of oregano was prepared at 5% (*w*/*v*) using dry oregano and water as a solvent; the solution was kept at 65 °C for 10 min, under magnetic agitation and further filtration. Commercial organic cranberry and pomegranate juices were used; the extract was filtered at 25 °C before its addition to the blend. The resveratrol solution at 5% (*w*/*v*) was prepared using water as a solvent at 25 °C, and then it was filtered. It was very important to obtain the extracts of cranberry, blueberry, beetroot, pomegranate and pitaya at 25 °C in order to preserve their antioxidants. The extracts were obtained in this manner because one of the main objectives of the study was to propose a cheap and simple method of getting the extracts, that does not involve complex leaching or extraction procedures that require using toxic solvents. The natural aqueous extracts and the film matrix solutions were compatible. For the quantification of antioxidants, Folin-Denis reagent (quality for determination of phenols) and gallic acid (97.5–102.5% titration) were purchased from Sigma-Aldrich (Toluca, Edo. Mex., Mexico), ethanol (99.9% purity) was purchased from Fermont (Monterrey, NL, Mexico) and sodium carbonate (anhydrous) was purchased from Merck (Naucalpan de Juarez, Edo. Mex., Mexico).

### 4.2. Synthesis of Films

The chitosan was dissolved in a glacial acetic acid solution (1% *v*/*v*) in order to prepare a chitosan solution at 2% (*w*/*v*). The starch solution (2% *w*/*v*) was prepared at 90 ± 2 °C for 20 min using constant stirring. The solution was kept at room temperature (25 °C approximately) for the films preparation. Each film was prepared by mixing 40 mL of chitosan solution with 40 mL of rice starch solution. After that, 0.2 mL of glycerol were added as plasticizer. Then, the extracts which contains natural antioxidants were added in three different quantities referred to the amount of the total weight of the chitosan-starch blend: 0.4, 1.6 and 4 mL (corresponding to 0.5%, 2% and 5% *v*/*v*, respectively). Finally, the mixture was stirred for 5–10 min and poured into a polystyrene tray [52]. The drying process took 15 days at controlled room temperature (25 °C). The experimental design consists of twenty-two films: one control film (QS2) and twenty-one films with natural extracts (three films for each extract). The complete experimental design and the film nomenclature are presented in Table 6. Three replications per sample were prepared. 

### 4.3. Evaluation of Films Coloration

The evaluation of film coloration was carried out visually on day 1 and compared with that observed on day 15, in order to understand the factors involved in the coloration changes and their relationship with other film properties. Furthermore, the coloration was evaluated by means of an analyzer software Image Color Summarizer (Canada) [53]. RGB values of each film were obtained through this software using photos taken on day 1 and day 15. During the image acquisition, each film was placed on a white background; the distance between the film and the camera was approximately 25 cm. The images were captured using a 5 megapixels camera at resolution of 480 × 800 pixels with Autofocus and CMOS image sensor, using two fluorescent lights for illumination in a closed lab room to avoid other light sources. All the images were saved and further processed using a personal computer and an image analyzer software. RGB is a color model based on additive synthesis (representation of a color by mixing of the three primary light colors: red (R), green (G) and blue (B). According to this model, red color is formed by RGB values of 255, 0, 0, while green and blue correspond to 0, 255, 0 and 0, 0, 255, respectively (monochromatic colors). The absence of color (black) is obtained when the values of RGB are 0, 0, 0. The yellow color is obtained with RGB values 255, 255, 0, the cyan color appears at RGB values 0, 255, 255 and the magenta color at RGB values 255, 0, 255 (intermediate colors). The white color is formed at the RGB maximum values 255, 255, 255 [16].

### 4.4 Apparent Density

This film property was measured according to the procedure used by Ahmed et al. [54]. Every film sample was cut in a circular shape and then it was weighed. Also, its diameter and height were measured. The apparent density (ρ) was obtained by the following Equation (1):

(1) $$\rho =\frac{W}{\left[\pi \;\times \;{\frac{D}{2}}^{2}\times \;H\right]\;}$$

where

- D = sample diameter
- H = sample height
- W = sample weight.

### 4.5 Fourier Transform Infrared Spectroscopy (FTIR)

The chitosan-starch films were analyzed by means of FTIR spectroscopy equipment (Perkin Elmer Spectrum 100, Perkin Elmer, Waltham, MA, USA) in order to determine the presence of functional groups and to elucidate interactions among the film compounds. The analysis was carried out using an attenuated total reflection (ATR) method, with a total of 12 scans and a resolution of 4 cm^−1^. FTIR spectra were recorded from 4000 cm^−1^ to 600 cm^−1^ in the wavenumber scale. 

### 4.6. Determination of Antioxidant Capacity of the Films

#### 4.6.1. Extraction

Based on a modification of the method published by Adom et al. [55], the natural antioxidants were gotten from 0.25 g of each film. Four milliliters of ethanol (99.9% purity) were added to each sample. This solvent was selected because it is compatible with the polar nature of the antioxidants. Afterwards, the samples were put in a water bath (Polystat 12050-00 Circulator, Cole-Parmer, Vernon Hills, IL, USA) at 25 °C and 90 rpm for 10 min. Then, the supernatant of each sample was centrifuged (Thermo Microlite-RF, Thermo Fisher Scientific, Waltham, MA, USA) at 19 °C and 13,000 rpm for 10 min. The procedure was repeated two times more. Finally, the extracts were stored and protected from light. 

#### 4.6.2. Qualification of Natural Antioxidants

Each extract was analyzed in triplicate using a UV-Vis spectrophotometer (Cintra 303, GBC Scientific Equipment, Mexico City, Mexico) with the purpose of detecting the presence of antioxidants. UV spectra were recorded within the wavelength interval ranging from 200 nm to 400 nm. The antioxidants were identified through their characteristic peaks: anthocyanins at 280 nm according to Lee et al. [22], thymol and carvacrol at 270 nm according to Amadio et al. [23], betalains and resveratrol at 280–350 nm according to Jaworska et al. [24]. 

#### 4.6.3. Antioxidant Capacity (Reducing Capacity)

A modification of the method used by Singleton et al. [56] was applied to determine the antioxidant capacity of each film. This modified method consisted in using 0.3 mL of film extract, 1.5 mL of Folin-Denis reagent (diluted 10 times in distilled water) and 1.2 mL of sodium carbonate (7.5% *w*/*v*) for analyzing each film. The samples were stirred for a few minutes, and then covered with parafilm and kept at room temperature for 30 min. Absorbance at 765 nm of each extract was measured in triplicate using a UV-Vis spectrophotometer (Cintra 303). The antioxidant capacity was expressed as mg/mg equivalent of gallic acid [22,57,58,59]. A calibration curve of gallic acid was performed using solutions (in 99.9% purity ethanol) at different concentrations (0, 40, 80, 120, 160 and 200 ppm) [60,61]. The antioxidant capacity was calculated through the following regression model (2):

y = 0.0006x + 0.2096 (R^2^ = 0.9971) (2)

### 4.7. Scanning Electronic Microscopy (SEM)

The films morphology was analyzed by means of a scanning electronic microscope (JEOL JSM 7100F, JEOL USA, Inc., Peabody, MA, USA) using magnifications of 500×, 750×, 1500× and 5000×. A carbon strip was used to hold each sample during the analysis. 

### 4.8. Thermogravimetric Analysis (TGA)

The thermal stability of the films was determined using a thermogravimetric analyzer (TA Instruments TGA Q500 V20.13 Build 39, TA Instruments, New Castle, DE, USA), under a temperature interval of 25–600 °C, a scan speed of 10 °C/min, sample weights of 15–22 mg and air as the purge gas. 

### 4.9. Statistical Analysis

Statistical evaluation presented in Table 1, Table 2, Table 3, Table 4 and Table 5 were performed using MS Excel (2013 version, Microsoft, Redmond, WA, USA). Multi-factor analysis of variance (ANOVA) using Minitab (16 version, Pennsylvania State University, State College, PA, USA) was used to determine if the type of extract, the type of antioxidant, the quantity of extract added to each film, or the interaction of these factors had a significant impact on the antioxidant capacity of the films. The statistically significant differences between means were evaluated using the Tukey’s test (α = 0.05).

## 5. Conclusions

Based on the results that were previously discussed, the addition of natural extracts gives chitosan-starch a higher apparent density values. According to the FTIR results, the only major difference among the film’ spectra is the intensity of the signals (% transmittance) and the width of the –OH band located at 3400–3300 cm^−1^. The FTIR spectra are similar, because both chitosan and starch have cyclic structures and multiple –OH bonds which are typical of saccharides, water, carboxylic acids, antioxidants, etc. The presence of the antioxidants in the films could be deduced by the observation of film coloration as well as the UV-Vis spectroscopy. From the results obtained in the UV-Vis analysis, it was confirmed that the films show characteristic peaks at 260–275 nm (hypochromic shifts were observed in all cases) indicating the presence of natural antioxidants in small amounts (there was no significant differences between the quantities). It is important to point out that the amount of extract (not of antioxidants) is related to antimicrobial properties. The amount of extracts does not have an influence on the film surface structure and the antimicrobial activity appears to be independent of the film surface structure. Based on the results obtained in the TGA analyses performed on some samples, a relationship between the amount of extract and the improvement in the film thermal properties was determined, and in particular, QSAm2, QSAm5 and QSO5 films proved to suffer the lowest weight losses among the studied samples, which implies that these films are the most thermally-stable and also reveals the preponderant role of anthocyanins and thymol-carvacrol. 

An important finding of this study was that improving the antimicrobial activity against mesophilic aerobic and coliforms bacteria and fungi [15] does not necessarily imply strong differences among the antioxidant capacities of the modified films. Moreover, the addition of natural extracts provided chitosan-starch films with better thermal and physical properties, making them suitable for food packaging applications, because they proved to be non-toxic, cheap and effective against micro-organisms and light. Although the type as well as the quantity of antioxidant have a significant effect on the final properties of the films, the results indicate that the influence exerted by the type of antioxidant was more substantial than that of the quantity that was added. Future research directions include a further study on the application of these films in food packaging and the sensory properties test. In addition, interesting research matter will come from looking for alternative antioxidant sources that are able to offer an optimum balance between sustainability, efficiency and applicability in terms of their antimicrobial, mechanical, optical, physical, morphological and thermal properties. 

## Figures and Tables

**Table 1:** Visual evaluation of film coloration and average RGB values at day 1 and 15.

Film ^(1)^	Coloration		Average RGB Day 1			Average RGB Day 15		
	Day 1	Day 15	Red	Green	Blue	Red	Green	Blue
QS2	Light yellow	Light yellow	159 ± 11.239	160 ± 11.532	158 ± 15.099	137 ± 45.28	140 ± 45.177	125 ± 48.445
QSA0.5	Slightly pink	Yellow	148 ± 11.239	148 ± 7.024	140 ± 9.165	153 ± 30.138	139 ± 28.827	89 ± 33.501
QSA2	Slightly pink	Light brown	146 ± 11.15	144 ± 7.767	137 ± 7.211	132 ± 24.986	94 ± 29.137	25 ± 31.764
QSA5	Pink	Reddish-brown	143 ± 18.52	140 ± 13.051	131 ± 4.725	122 ± 40.501	54 ± 45.236	16 ± 41.218
QSAm0.5	Slightly brown	Yellow	150 ± 10.0167	150 ± 10.0167	139 ± 9.539	157 ± 76.839	109 ± 78.619	29 ± 80.894
QSAm2	Light brown	Light brown	145 ± 31.214	142 ± 32.036	130 ± 33.645	103 ± 53.799	42 ± 34.559	26 ± 29.67
QSAm5	Brown	Reddish-brown	144 ± 30.643	136 ± 39.803	120 ± 47.721	88 ± 60.002	39 ± 51.643	27 ± 49.729
QSB0.5	Pink	Yellow	166 ± 14.012	70 ± 25.515	85 ± 24.758	135 ± 20.404	119 ± 29.67	69 ± 4.509
QSB2	Pinkish-red	Brown	146 ± 13.748	33 ± 35.218	47 ± 32.516	136 ± 31.973	92 ± 37.634	26 ± 38.314
QSB5	Wine red	Reddish-brown	99 ± 27.622	23 ± 30.643	20 ± 31.005	108 ± 31.973	52 ± 37.634	21 ± 38.314
QSG0.5	Slightly yellow	Light yellow	152 ± 12.055	153 ± 7	145 ± 9.504	150 ± 32.959	136 ± 36.115	86 ± 46.09
QSG2	Light pink	Light brown	157 ± 9.504	147 ± 9.073	136 ± 9.073	136 ± 29.137	92 ± 39.715	23 ± 42.532
QSG5	Pink	Reddish-brown	155 ± 14.295	146 ± 10	135 ± 8.544	108 ± 31.214	37 ± 39.576	19 ± 43.143
QSO0.5	Slightly yellow	Light yellow	157 ± 18.877	157 ± 18.77	146 ± 17.691	148 ± 22.912	146 ± 22.185	130 ± 26.083
QSO2	Light yellow	Light brown	147 ± 25.515	145 ± 26.058	133 ± 24.515	128 ± 18.175	123 ± 19.425	98 ± 26.633
QSO5	Yellow	Light brown	139 ± 23.065	133 ± 23.007	109 ± 25.514	131 ± 17.473	122 ± 13	86 ± 6.506
QSP0.5	Pink	Brown	148 ± 14	146 ± 8.505	136 ± 8.082	150 ± 13.051	79 ± 19.008	22 ± 16.093
QSP2	Pinkish-red	Reddish-brown	148 ± 17.039	145 ± 14.12	139 ± 13.428	115 ± 23.544	44 ± 34.597	28 ± 32.129
QSP5	Fuchsia-red	Dark red	147 ± 20.008	146 ± 14.012	141 ± 10.692	71 ± 38.436	31 ± 36.846	29 ± 36.295
QSR0.5	Slightly yellow	Light yellow	156 ± 19.757	156 ± 20.297	154 ± 19.218	135 ± 32.078	136 ± 35.921	121 ± 47.57
QSR2	Slightly brown	Light brown	147 ± 15.275	148 ± 15.716	143 ± 15.275	140 ± 40.951	136 ± 43.108	116 ± 47.71
QSR5	Slightly brown	Light brown	150 ± 15.822	151 ± 15.395	146 ± 15.947	134 ± 38.423	131 ± 40.501	107 ± 49.426

^(1)^ Where: Q = chitosan, S = starch, A = cranberry, Am =blueberry, B = beetroot, G = pomegranate, O = oregano, P = pitaya/dragon fruit, R= resveratrol; numbers mean the weight percentage of extract used in each film.

**Table 2:** Apparent density values of the films.

Sample	Apparent Density (g/cm^3^)	Sample	Apparent Density (g/cm^3^)
QS2	0.0835 ± 0.0001	QSG2	0.0713 ± 0.0002
QSA0.5	0.1034 ± 0.00004	QSG5	0.1087 ± 0.0002
QSA2	0.0933 ± 0.00035	QSO0.5	0.0757 ± 0.0002
QSA5	0.1051 ± 0.000055	QSO2	0.0677 ± 0.00007
QSAm0.5	0.0908 ± 0.00031	QSO5	0.0641 ± 0.0002
QSAm2	0.0916 ± 0.0009	QSP0.5	0.0711 ± 0.0001
QSAm5	0.0847 ± 0.00044	QSP2	0.0752 ± 0.00004
QSB0.5	0.0907 ± 0.0005	QSP5	0.0646 ± 0.0002
QSB2	0.0754 ± 0.00039	QSR0.5	0.0649 ± 0.00007
QSB5	0.0814 ± 0.00039	QSR2	0.0816 ± 0.00003
QSG0.5	0.0984 ± 0.00026	QSR5	0.0807 ± 0.00009

**Table 3:** Wavelength of antioxidant characteristic peaks for each film.

Film	Wavelength, nm	Film	Wavelength, nm
QS2	------	QSG2	264.32 ± 0
QSA0.5	274.99 ± 1.48	QSG5	264.89 ± 2.10
QSA2	275.70 ± 1.23	QSO0.5	269.16 ± 3.58
QSA5	273.71 ± 1.54	QSO2	262.90 ± 0.65
QSAm0.5	262.61 ± 0.74	QSO5	262.47 ± 1.92
QSAm2	267.59 ± 0.49	QSP0.5	266.74 ± 0.98
QSAm5	273.14 ± 5.16	QSP2	267.31 ± 0
QSB0.5	261.76 ± 0.74	QSP5	261.48 ± 2.46
QSB2	267.73 ± 1.13	QSR0.5	262.76 ± 0.25
QSB5	262.33 ± 1.07	QSR2	267.73 ± 0.43
QSG0.5	265.46 ± 1.72	QSR5	267.31 ± 0

**Table 4:** Antioxidant capacity of each film (mg/mg equivalent to gallic acid).

Film	Antioxidant Capacity, mg/mg	Film	Antioxidant Capacity, mg/mg
QS2	------	QSG2	0.20984 ± 2.64 × 10^−6^
QSA0.5	0.21001 ± 4.85 × 10^−5^	QSG5	0.20997 ± 2.15 × 10^−5^
QSA2	0.20991 ± 1.61 × 10^−5^	QSO0.5	0.21003 ± 2.73 × 10^−5^
QSA5	0.20992 ± 6.65 × 10^−5^	QSO2	0.21020 ± 1.46 × 10^−4^
QSAm0.5	0.20998 ± 6.23 × 10^−5^	QSO5	0.21003 ± 6.92 × 10^−6^
QSAm2	0.20997 ± 4.69 × 10^−5^	QSP0.5	0.20997 ± 3.48 × 10^−6^
QSAm5	0.20999 ± 6.37 × 10^−5^	QSP2	0.21002 ± 4.06 × 10^−5^
QSB0.5	0.20994 ± 9.18 × 10^−5^	QSP5	0.21008 ± 6.67 × 10^−5^
QSB2	0.21004 ± 7.27 × 10^−6^	QSR0.5	0.20995 ± 5.63 × 10^−5^
QSB5	0.20998 ± 9.18 × 10^−5^	QSR2	0.21005 ± 5.82 × 10^−6^
QSG0.5	0.21003 ± 2.73 × 10^−5^	QSR5	0.21005 ± 5.58 × 10^−5^

**Table 5:** Weight loss percentage at 135°, 320° and 600 °C of each film.

Sample	Weight Loss Percentage at 135 °C	Weight Loss Percentage at 320 °C	Weight Loss Percentage at 600 °C
QSAm2	10.208 ± 0.2	57.712 ± 0.2	90.52 ± 0.2
QSAm5	9.812 ± 0.2	53.782 ± 0.2	88.571 ± 0.2
QSB2	8.264 ± 0.2	55.290 ± 0.2	96.519 ± 0.2
QSB5	7.212 ± 0.2	51.424 ± 0.2	97.465 ± 0.2
QSO5	17.920 ± 0.2	65.787 ± 0.2	90.507 ± 0.2
QSP0.5	9.216 ± 0.2	59.783 ± 0.2	96.054 ± 0.2

**Table 6:** Experimental design of chitosan-starch films with natural antioxidants.

Number	Sample ^(1)^	Number	Sample ^(1)^
1	QS2	12	QSG2
2	QSA0.5	13	QSG5
3	QSA2	14	QSO0.5
4	QSA5	15	QSO2
5	QSAm0.5	16	QSO5
6	QSAm2	17	QSP0.5
7	QSAm5	18	QSP2
8	QSB0.5	19	QSP5
9	QSB2	20	QSR0.5
10	QSB5	21	QSR2
11	QSG0.5	22	QSR5

^(1)^ Where: Q = chitosan, S = starch, A = cranberry, Am =blueberry, B = beetroot, G = pomegranate, O = oregano, P = pitaya/dragon fruit, R= resveratrol; numbers mean the weight percentage of extract used in each film.
